# Use of QSPR Modeling to Characterize *In Vitro* Binding of Drugs to a Gut-Restricted Polymer

**DOI:** 10.1007/s11095-018-2356-y

**Published:** 2018-03-08

**Authors:** Christine Taylor Brew, James F. Blake, Anita Mistry, Fengling Liu, Diana Carreno, Deidre Madsen, YongQi Mu, Martha Mayo, Wilhelm Stahl, David Matthews, Derek Maclean, Steve Harrison

**Affiliations:** 1 Research Department, Relypsa, Inc., a Vifor Pharma Group Company, 100 Cardinal Way, Redwood City, California 94063 USA; 2 Computational Chemistry Department, Array BioPharma Inc, Boulder, Colorado USA; 3 Clinical Development, Relypsa, Inc., a Vifor Pharma Group Company, Redwood City, California USA; 4 Technical Operations, Relypsa, Inc., a Vifor Pharma Group Company, Redwood City, California USA

**Keywords:** drug interaction, electron affinity, hydrogen bonding, ionization potential, lipophilicity

## Abstract

**Purpose:**

Polymeric drugs, including patiromer (Veltassa®), bind target molecules or ions in the gut, allowing fecal elimination. Non-absorbed insoluble polymers, like patiromer, avoid common systemic drug-drug interactions (DDIs). However, the potential for DDI via polymer binding to orally administered drugs during transit of the gastrointestinal tract remains. Here we elucidate the properties correlated with drug-patiromer binding using quantitative structure-property relationship (QSPR) models.

**Methods:**

We selected 28 drugs to evaluate for binding to patiromer *in vitro* over a range of pH and ionic conditions intended to mimic the gut environment. Using this *in vitro* data, we developed QSPR models using step-wise linear regression and analyzed over 100 physiochemical drug descriptors.

**Results:**

Four descriptors emerged that account for ~70% of patiromer-drug binding *in vitro*: the computed surface area of hydrogen bond accepting atoms, ionization potential, electron affinity, and lipophilicity (*R*^*2*^ = 0.7, *Q*^*2*^ = 0.6). Further, certain molecular properties are shared by nonbinding, weak, or strong binding compounds.

**Conclusions:**

These findings offer insight into drivers of *in vitro* binding to patiromer and describe a useful approach for assessing potential drug-binding risk of investigational polymeric drugs.

**Electronic supplementary material:**

The online version of this article (10.1007/s11095-018-2356-y) contains supplementary material, which is available to authorized users.

## Introduction

Large polymer drugs typically have a high density of binding sites and thus a large capacity to absorb their target species (1). As large insoluble particles (>10 μm), they are excreted, along with the bound material, rather than being digested and absorbed into the blood stream. This has the advantage of reducing the potential for systemic off-target effects or drug-drug interactions (DDIs), such as cytochrome P450 enzyme inhibition. However, the potential for DDIs during transit of the gastrointestinal tract remains.

**Fig. 1:**
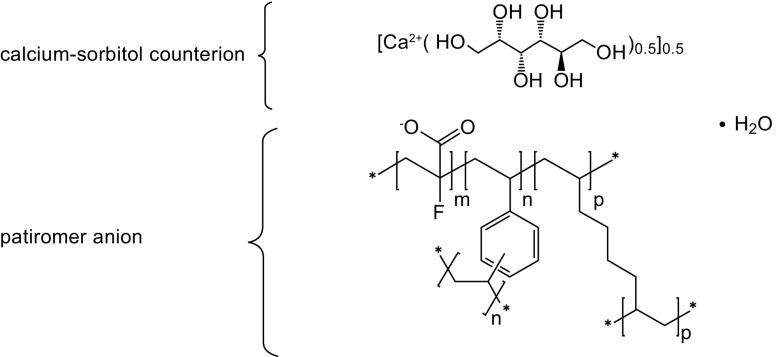
Chemical structure of patiromer sorbitex calcium. m = number of 2-flouro-2-propenoate groups. n, p = number of crosslinking groups. •H_2_O = Associated water. ^*^ = Indicates an extended polymeric network. m = 0.91. n + *p* = 0.09. Image reproduced with permission from Relypsa, Inc. (2).

Veltassa® (patiromer; Relypsa, Inc., a Vifor Pharma Group Company; Redwood City, CA) is a non-absorbed, potassium binding drug approved for the treatment of hyperkalemia (elevated serum potassium). The active ingredient is patiromer sorbitex calcium which consists of the active moiety, patiromer, a cross-linked anionic polymer, and a calcium sorbitol counterion (Fig. 1) (2). It is formulated as a powder that is mixed with water and given orally, once daily at a starting dose of 8.4 g. Patiromer binds potassium in the gastrointestinal tract, particularly the colon. Results of an *in vitro* study showed that patiromer has a binding capacity of 8.5–8.8 mEq of potassium per gram of polymer (3). Patiromer, along with bound potassium, is eliminated in the feces, reducing serum potassium levels (3).

In addition to binding potassium during transit of the gut, there is the potential for negatively charged patiromer to bind to co-administered oral drugs, particularly those that are positively charged or that bind its counter-ion, calcium. It is important to understand the clinical potential for patiromer-drug interactions, as patients with hyperkalemia typically suffer from chronic disease, notably chronic kidney disease, heart failure, and diabetes, and may have multiple comorbid conditions.

As previously reported, compounds tested for DDIs in human subjects were prescreened for the ability to bind patiromer *in vitro* (4). Significant binding to patiromer (>30% of drug bound) was observed with 14 of the 28 small molecule drugs assessed and this formed the basis for compound selection in the clinical studies. In this clinical testing, 3 of the 12 compounds tested showed a potential for clinically relevant DDIs when administered together with patiromer; however, no clinically meaningful DDIs were observed when the drugs were given 3 h apart (4).

Of necessity, clinical DDI studies are incomplete, as only a subset of drugs that might be co-administered can be tested. A limited literature exists on polymer DDIs; however, the unique engineered properties of each polymer make it unlikely that findings with one can be generalized to another. Rather, *in vitro* assays and in silico modeling can be used to help predict those compounds most or least likely to bind a given polymer. For example, Walker and colleagues (5) reported the development of a quantitative structure-property relationship (QSPR) model describing binding *in vitro* to the polymer bile acid sequestrant, colesevelam hydrochloride. Using partial least-squares regression analysis, drug lipophilicity emerged as the primary determinant of colesevelam-drug binding. The authors also went on to show that *in vitro* binding sensitively identified compounds with a low probability of causing a clinically significant DDI *in vivo* (5).

Here we report the development of QSPR models of patiromer-drug binding *in vitro* based on step-wise linear regression analysis of over 100 measured and calculated drug properties and *in vitro* binding data (4). We identify the physicochemical characteristics of drugs that best predict binding to patiromer *in vitro*, as well as the molecular properties of drugs with strong, weak, or no binding to patiromer. The QSPR models reported here illustrate the utility of an in silico approach to evaluate the potential for drugs to bind polymeric drugs.

## Materials and Methods

### Test Articles

#### Patiromer

Patiromer sorbitex calcium is manufactured by Relypsa, Inc., under good manufacturing practices.

#### Drugs

All test drugs were obtained from US Pharmacopeia (Rockville, MD), except rivaroxaban, cinacalcet hydrochloride (TRC; North York, ON, Canada) and apixaban (Alsachim; Strasbourg, France).

### pKa, LogP and LogD Determination

Test drug properties pKa, LogP, and LogD, as shown in Table I, were experimentally determined as follows.

**Table I:** Physicochemical Properties of Test Compounds and *In Vitro* Patiromer Binding

	Properties				*In Vitro* Binding (% Drug Recovered)			
Name	BCS Class	pKa	LogP	LogD pH 3.0, 4.5, 5.9	*Conc Used* *In Vitro* (μg/mL)	SGF (pH 3)	Acetate Buffer (pH 4.5)	SIF (pH 5.9)
No Significant Binding ≥70% drug recovered (ie, ≤30% drug bound) in all tested buffers								
Allopurinol	III	9.18 A 12.12 A	−0.3 (N)	−0.3 (all)	100	85.7	90.1	94.2
Amoxicillin	III	2.60 A 7.35 B 9.59 A	−0.02 (N) 2.19 (C)	1.65 0.46 0.00	500	ND^a^	99.2	99.4
Apixaban	III	NA	<1.40 (N)	<1.40 (all)	5	75.6	97.3	97.8
Aspirin	III	3.50 A	0.90 (N)	0.78 −0.14 −1.50	81	100.7	99.8	99.9
Atorvastatin calcium	II	4.47 A	4.01 (N) 1.04 (A)	3.99 3.69 2.58	10	91.2	93.3	101.1
Cephalexin	III	2.56 A 7.10 B	−1.05 (N)	−1.20 −1.06 −1.08	250	88.7	95	104.4
Digoxin	II	NA	1.64 (N)	1.64 (all)	0.125	ND^a^	109.3	103.4
Glipizide	II	5.06 A	2.91 (N) −0.45 (A)	2.91 2.82 2.08	5	72.9	96.5	98.5
Lisinopril	III	1.63 A 3.13 A 7.13 B 10.75 B	−0.51 (N)	−0.85 −0.52 −0.54	5	77.9	100.7	98.5
Phenytoin sodium	II	8.18 A	2.43 (N) −0.09 (A)	2.43 (all)	25	83.4	90.2	92.8
Riboflavin	I	9.87 A	<−1.50 (C)	<−1.50 (all)	1.2	95.6	ND^b^	96.5
Rivaroxaban	II	NA	1.43 (N)	1.43 (all)	10	71.9	92.9	95.1
Spironolactone	II	NA	2.53 (N)	2.53 (all)	25	78.7	98.6	96.8
Valsartan	II	3.73 A 4.4 A	3.98 (N) 1.48 (A)	3.9 2.83 0.46	40	86.4	101.4	98.0
Weak Binding 45–70% drug recovered (30–55% drug bound) in one test media only								
Clopidogrel bisulfate	II	4.66 B	4.06 (N) 0.95 (C)	2.40 3.67 4.03	75	66	ND^a^	ND^a^
Furosemide	IV	3.62 A 10.16 A	2.20 (N) −0.82 (A)	2.10 1.25 −0.03	20, 10 (SGF)	67.3	94.5	79.1
Lithium carbonate*	I	NA	NA	NA	600	93.3	88.8	56.9
Metformin HCl	III	2.94 B 13.7 B	<−1.50 (N)	<−1.50 (all)	500	48.9	81.8	80.2
Metoprolol tartrate	I	9.61 B	1.91 (N)	−4.70 −3.20 −1.80	25	71.7	85.9	68.6
Verapamil HCl	I	8.95 B	4.20 (N) 0.52 (C)	0.52 0.59 1.24	120	51.7	88.6	77.9
Warfarin (R/S)	II	4.94 A	3.25 (N) −0.77 (A)	3.25 3.12 2.25	2	66.3	92.5	97.3
Strong Binding ≤45% drug recovered (≥55% bound) in 2 or more test media								
Amlodipine besylate	I	9.21B	3.39 (N) 1.44 (C)	1.44 1.44 1.46	2.5	10.8	36.6	13.1
Cinacalcet HCl	IV	8.85 B	5.58 (N) 2.41 (C)	2.41 2.44 2.83	30	13.4	19.3	18.3
Ciprofloxacin HCl	IV	6.35 A 8.33 B	−0.27 (N)	−3.75 −2.25 −0.98	250, 100 (SIF)	18	24.8	6.9
Quinidine	I	4.39 B 9.06 B	3.75 (N) 0.85 (C)	−0.07 0.63 1.03	300	12.6	43.1	24.3
Thiamin*	III	4.88 B	<−1.50 (C)	<−1.50 (all)	1	28.8	50.7	42.9
Trimethoprim	II	7.14 B	0.78 (N)	−3.36 −1.87 −0.49	100	36.3	55.3	28.4
Could not be tested *in vitro*								
Levothyroxine sodium*	II	2.00 A 6.65 A 8.73 B	3.44 (N)	3.44 3.44 3.37	NA	ND^a^	ND^a^	ND^a^

Values shown are % test drug recovered following co-incubation in the specified buffer

Legend: A = acid, B = base, (A) = anionic, (C) = cationic, (N) = neutral

An asterisk * designates compounds not included in subsequent QSPR model building, due to limitations of computational methodology or instability

^a^Drug could not be tested in the indicated condition due to instability; ^b^Subject to lysis in acetate buffer

BCS, Biopharmaceutics Classification System; Conc, concentration; HCl; hydrochloride; NA, not applicable; ND, not determined; QSPR, quantitative structure-property relationship; SGF, simulated gastric fluid; SIF, simulated intestinal fluid

#### pKa

Test drug pKas were determined experimentally by titration in aqueous solution. Ionization state was monitored by UV or pH. Reported values are the average of three independent determinations. For drugs with poor aqueous solubility, which required a co-solvent (25 to 50%), pKa was determined by Yasuda-Shedlovsky extrapolation. Drug concentration was in the range 17–31 μM (UV) or ≥0.4 mM (pH).

#### logP

Compound logPs were determined by titration in various ratios of octanol and water at concentrations no lower than 0.4 mM. The shift of the aqueous pKas in the presence of octanol was used to determine the logP of the different species.

#### logD(7.4)

The logD of the sample was determined at pH 7.4 by liquid-liquid distribution chromatography. Compounds were eluted from an octanol-coated column with an octanol-saturated mobile phase adjusted to pH 7.4. Compound retention time was converted to logD value by comparison to the retention time of a set of standard compounds with well-characterized logD octanol values.

### *In Vitro* Binding Assays

Patiromer binding to test compounds was assessed by comparing free test drug concentrations after incubation with and without patiromer. Patiromer anion was added to a concentration of 25.2 g/L, representing a maximal patiromer dose dispersed in the approximate volume of fluid in the upper adult gastrointestinal tract (1 L) (6). Test drug concentrations were based on the lowest clinically relevant dose of test drug in US prescribing information in a 1-L volume. Test drug stock solutions were prepared at 5 mg/mL (6 mg/mL for lithium carbonate) in either dimethyl sulfoxide or Milli-Q water and diluted to the final desired concentration in test media. Drugs that were not soluble at concentrations equivalent to the lowest prescribed dose (ciprofloxacin, furosemide, phenytoin) were used at the maximum achievable concentration (see Table I for final values).

Three test buffers were used: simulated gastric fluid without added pepsin (SGF; initial pH 1.2), acetate buffer (AB; initial pH 4.5), and simulated intestinal fluid without added pancreatin (SIF; initial pH 6.8). Tween-20 was included at 0.05% *v*/v as a surfactant to aid in solubilizing test drugs. The final pH of the media with patiromer added at 25.2 g/L was as follows: SGF pH 3.0, AB pH 4.6, and SIF pH 5.9.

Binding studies were performed in a 10 ml volume in 16×100 mm borosilicate tubes, and incubated for 3 h at 37°C with end-over-end rotation at ~25 rpm. After incubation, patiromer was allowed to settle for 5 min; supernatants were withdrawn and passed through a 0.45 μm filter, collected by centrifugation, and transferred to chromatography vials for analysis. Control samples containing buffer and drug, but without patiromer, were subjected to the same experimental process. For 24 of the test drugs, a reversed phase high performance liquid chromatography method was used for analysis in the three test matrices. Atorvastatin and digoxin were measured using liquid chromatography-mass spectrometry methods, while lithium was analyzed using ion chromatography. All analytical methods were qualified to demonstrate specificity, linearity, accuracy and precision, and stability. During method qualification, we conducted a pretest of each drug in test media alone to assure no significant loss in recovery during the incubation or filtration steps. If there was not sufficient recovery compared to theoretical concentration, the drug could not be evaluated in that medium. The recovered drug in the presence or absence of patiromer was used to calculate percent recovery of test drug, and represents the geometric mean of 12 replicates.

Dose-response studies were performed using the same method across a range of test drug concentrations.

### Drug Structures and Properties

A total of 121 computed molecular descriptors were considered in the development of the QSPR models, including electronic, steric, topological, and hydrophobic terms (**Supplementary Table** **SI**).

The molecular structure of each drug listed was downloaded from the Division of Specialized Information Services website of the National Laboratory of Medicine (http://chem.sis.nlm.nih.gov/chemidplus/) and processed to produce an appropriate three-dimensional representation with the LigPrep 3.0 application in Schrödinger suite, accessible from the Maestro interface (QikProp; Schrödinger, LLC; 2014). The LigPrep 3.0 program used the following criteria: neutral charge state, generate possible tautomers, generate conformers, force field: OPLS_2005. A final energy minimization was carried out with the OPLS_2005 force field, with implicit solvent (water).

Molecular properties were computed with several programs: various charged partial surface area terms were computed with the QikProp 3.9 program (using the –altclass option; QikProp; Schrödinger, LLC; 2014); ACD Labs logD 12.00 (Advanced Chemistry Development, Inc.; 2014); BioByte ClogP 4.9 (BioByte Corp); and an in-house program (polar version 1.1) (7). The polar program utilized the AM1 semi-empirical Hamiltonian to compute the molecular charges and molecular orbital energies. As some of the test compounds may be ionized at various pH levels, logD values were computed [logD = logP - log(1+ 10^pKa - pH^)], at various pH values (pH = 1.2, 3.0, 4.5, 5.9, 6.8). All statistics were performed with the R program 3.1.0 and standard statistical modules therein (8).

### QSPR Modeling

Step-wise linear regression was used to develop QSPR models describing properties driving drug-patiromer *in vitro* binding in each of the three test media used. For each drug evaluated by QSPR modeling, the mean geometric ratio of recovered drug with and without patiromer in each buffer was calculated and used in the subsequent regression analysis, along with the molecular properties shown in **Supplementary Table** **SI**.

Most of the molecular parameters used in model building were computed based on the neutral species, as required in QikProp. For this reason, lithium and thiamin were not included in the regression analysis. For factors that vary with pH (eg, LogD, pKa), the relevant values were included in the modeling of patiromer binding at that pH. Levothyroxine was not included in the regression analysis because *in vitro* binding could not be measured due to precipitation of levothyroxine in the presence of the patiromer calcium counter-ion.

The *R*^*2*^ value was calculated for each QSPR equation. In order to assess the accuracy of each linear regression model, a leave-one-out cross-validation was performed and the results were reported as a *Q*^*2*^ value (9). In addition, *p*-values for coefficients in the QSPR equations were calculated.

## Results

We selected 28 drugs to evaluate for binding to patiromer *in vitro* over a range of pH and ionic conditions intended to mimic the gut environment. Test drugs were initially selected based on relevance to the patiromer patient population and represented a chemically and functionally diverse group. The test panel included compounds with varied physicochemical properties from all four of the Biopharmaceutics Classification System (BCS) drug classes (Table I). Drugs that might be more likely to interact with patiromer due to positive charge, basicity, or hydrophilicity were also included in the test panel, as were several drugs with a narrow therapeutic index, as it is particularly important to be able to control the exposure of these types of therapeutics.

Values for pKa, logD and logP and *in vitro* binding data at three different pHs were determined experimentally (Table I). To measure binding, each compound was co-incubated with patiromer using the highest single clinical dose of polymer and the minimum clinical dose of test drug. This approach is expected to maximize the availability of drug binding sites on patiromer and the ability to detect drug-patiromer interaction. A 3-h co-incubation time was selected, as that is anticipated to be the longest time that the polymer and the co-administered drug will routinely be in close contact prior to absorption of the co-administered drug. Stomach exposure (SGF conditions) is less than 3 h (10) and upper GI exposure (AB buffer conditions) is about 3 h (11,12). The majority of tested drugs have a Tmax of less than 3 h (4), and little drug will be present in the colon (SIF conditions). Following co-incubation, patiromer and any bound drug were removed by centrifugation and the remaining unbound (recovered) drug was measured in the supernatant.

As shown in Table I, 14 of the 28 drugs tested (50%) did not show significant binding to patiromer, which was defined as ≥70% of the drug being recovered in the supernatant following co-incubation. Thirteen other compounds showed significant binding to patiromer: 8 drugs were weak binders, with 30–55% of drug bound to patiromer in a single test condition; 5 were strong binders, with >55% of drug bound in multiple test conditions. Levothyroxine, a known calcium binder, (13) could not be tested as it precipitated in the presence of patiromer and its calcium counter-ion. Four other drugs in the panel (clopidogrel, amoxicillin, digoxin, and riboflavin) could not be assayed in all buffer conditions due to instability (see Table I footnotes).

We investigated whether the *in vitro* binding data could be used to generate a QSPR that might allow in silico prediction of the potential of an untested drug to bind to patiromer. As the *in vitro* binding of each drug was assessed at a single concentration, and that concentration depended on the clinically relevant dose for each drug, we sought to demonstrate that the resulting binding was not strongly dependent on the tested concentration of drug. Consequently, we conducted further *in vitro* testing of patiromer’s interaction with three of the strong binders, amlodipine besylate, quinidine gluconate and trimethoprim, across a range of drug concentrations to determine the impact of drug concentration on binding (**Supplementary Figure** **S1**). Binding to patiromer did not show strong concentration dependence across a clinically relevant dose range for the three drugs tested. Thus, despite the varied concentrations of drug used in the *in vitro* studies, the data were judged suitable to develop in silico models for patiromer-drug binding.

Having confirmed that the test panel included a well-balanced set of binding and nonbinding drugs, data from 25 of the original 28 compounds were used to build QSPR models of patiromer-drug binding *in vitro*. The data for patiromer binding to the test drugs reported in Table I do not suggest a simple relationship between *in vitro* binding and any single structural feature or physical property of the compounds, such as lipophilicity, charge, or polarity. To build predictive models, we measured or calculated 121 physical property descriptors for the drugs (**Supplementary Table** **SI**; see Wessel *et al*. [7] for more on these descriptors) and applied step-wise linear regression with both forward and backward search selection to produce a model of patiromer-drug binding in each buffer. The resulting QSPR models were validated using the leave-one-out method, following an approach similar to that used by Walker *et al*. (2009) to define QSPR models for binding to colesevelam (5).

The QSPR model terms identified for patiromer-drug binding *in vitro* are given below and the corresponding coefficients and *p* values are reported in Table II**.**

**Table II:** Summary of QSPR Model Regression Coefficients and *P*-Values

Model	Intercept	SAAA1	IP.eV	EA.eV	QPlogPw	X.noncon	X.amide
SGF	−396.3906 (0.0000)	NA	46.4826 (0.0000)	NA	4.0192 (0.0005)	−2.1771 (0.0288)	−22.2272 (0.0659)
AB	−189.9966 (0.0052)	0.3818 (0.0001)	26.5812 (0.0012)	−16.8010 (0.0274)	NA	−1.6017 (0.0070)	NA
SIF	−294.4202 (0.0009)	0.4371 (0.0002)	36.8472 (0.0004)	−13.3460 (0.1402)	NA	−2.2171 (0.0036)	NA

Values in parentheses represent *p*-values from the linear regression model; see definition of terms, below

Coefficients:

SAAA1, sum of surface area on acceptor atoms (oxygen, nitrogen) Å^2^

IP.eV, PM3 calculated ionization potential (negative of HOMO energy)

EA.eV, PM3 calculated electron affinity (negative of LUMO energy)

QPlogPw, QikProp predicted water/gas partition coefficient

X.noncon, number of ring atoms not able to form conjugated aromatic systems (eg, sp^3^C)

X.amide, number of non-conjugated amide groups

AB, acetate buffer; NA, not applicable; QSPR, quantitative structure-property relationship; SGF, simulated gastric fluid; SIF, simulated intestinal fluid

In SGF buffer (pH 3.0), binding was best predicted by the physical parameters:

$$ \mathrm{IP}.\mathrm{eV}+\mathrm{QPlogPw}+\mathrm{X}.\mathrm{noncon}+\mathrm{X}.\mathrm{amide} $$

where IP.eV is the ionization potential, QPlogPw is the predicted partition coefficient in water/gas, X.noncon is the number of ring atoms not able to form conjugated aromatic systems, and X.amide is the number of non-conjugated amide groups.

The QSPR models for binding in SIF (pH 4.6) and AB (pH 5.9) test media also incorporate the ionization potential and X.noncon terms. However, two other terms appear, yielding the equation:

$$ \mathrm{SAAA}1+\mathrm{IP}.\mathrm{eV}+\mathrm{X}.\mathrm{noncon}+\mathrm{EA}.\mathrm{eV} $$

where SAAA1 is the computed surface area of hydrogen bond acceptor atoms and EA.eV is electron affinity.

**Fig. 2:**
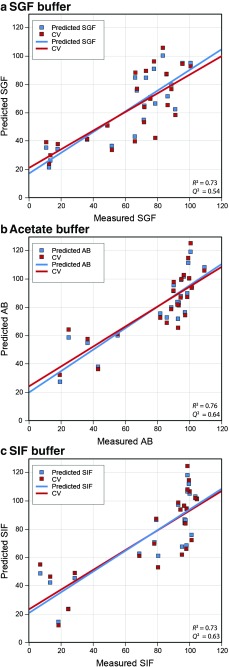
(a-c) Measured vs. predicted values for drug-patiromer binding derived from QSPR equations. Both the model (training set), and leave-one-out cross-validation set (CV set) are plotted, along with the best-fit line to the training set for each buffer. AB, acetate buffer; CV, cross-validation; QSPR, quantitative structure-property relationship; SGF, simulated gastric fluid; SIF, simulated intestinal fluid.

Predicted binding to patiromer was calculated for each drug using the relevant QSPR equation and compared to the experimentally observed values, as shown graphically in Fig. 2 and detailed in **Supplementary Table** **SII**. All of the models yielded a traditional *R*^*2*^ of ~0.7. A cross-validated *Q*^*2*^ value was generated using leave-one-out analysis. Q^2^ values of ~0.6 were obtained. An *R*^*2*^ of 0.7 implies that the variables in the model together account for ~70% of the variation in the binding to patiromer under the specified *in vitro* conditions. The remaining 30% variance is likely attributable to untested variables and to variability inherent in the assay system. The small difference between *R*^*2*^ and *Q*^*2*^ values (ie, *R*^*2*^ - *Q*^*2*^ < 0.3) suggests that our models are not over-fit and should be predictive for compounds that have not been included in the current study (14).

To further characterize the influence of the terms identified in the QSPR models on patiromer-drug binding *in vitro*, we compared the properties of the strong, weak, and non-binding compound groups identified earlier (Table III). The non-binding test compounds typically have multiple polar functional groups, such as cephalexin and riboflavin; or acidic groups, such as valsartan. Drugs that bound patiromer *in vitro* tended to have fewer polar groups and were more lipophilic than the nonbinders. Weak binders of patiromer, such as clopidogrel or metoprolol, were neutral to weakly basic, with limited polar functionality. Strong patiromer binding was seen *in vitro* with drugs containing basic amines not balanced by a nearby polar group, such as cinacalcet.

**Table III:** Patiromer Binding *In Vitro*, Molecular Properties Affecting Binding, and Observed Clinical DDI Results (4)

Binding categories	Test drug	Level of *in vitro* interaction with patiromer	QSPR molecular properties	AUC reduced when co-administration (4) (lower bound of the 90% CI <80%)
No binding	Allopurinol Amoxicillin Apixaban Aspirin Atorvastatin Cephalexin Digoxin Glipizide Lisinopril Phenytoin Riboflavin Rivaroxaban Spironolactone Valsartan	<30% drug bound in any test matrix^14^	Non-binding compounds tend to be highly polar with multiple polar functional groups (eg, nitro, sulfonamide, etc.). Compounds with acidic groups also show no binding	Not tested: no binding *in vitro*
Weak binder	Clopidogrel Furosemide Lithium Metformin Metoprolol Verapamil Warfarin (−R, −S)	30–55% bound in one test matrix	Weak *in vitro* drug binding to patiromer begins to occur as the number of polar groups on the test drug decreases or its lipophilicity increases. Weak binders of patiromer tend to be neutral to weakly basic, with limited polar functionality, such as clopidogrel or metoprolol	Metformin, 1000 mg
Strong binder	Amlodipine Cinacalcet Ciprofloxacin Levothyroxine** Thiamin* Trimethoprim Quinidine*	>55% bound in two or three test matrices	The strongest *in vitro* binding to patiromer is predicted to occur with drugs that contain basic amines that are not balanced by a polar functionality, eg, a carboxylate or cyano group or multiple amides, in close proximity to the basic group.	Ciprofloxacin, 500 mg Levothyroxine, 0.6 mg

* *In vitro* binders not included in clinical study; **Not tested *in vitro*

AUC, area under the plasma concentration–time curve from 0 to infinity; CI, confidence interval; DDI, drug-drug interaction; QSPR, quantitative structure-property relationship

Thus, in this manuscript, we have demonstrated that the QSPR models predicted *in vitro* binding. However, as published previously, *in vitro* binding was not predictive of *in vivo* DDIs (4).

## Discussion

A QSPR model has been used to illustrate an in silico approach to evaluate potential binding of drugs to polymer therapeutics. Such QSPR models can be developed based on a subset of possible interacting drugs, and can reveal the molecular characteristics most predictive of *in vitro* binding to polymers, as evidenced by the close correlation between predicted and observed binding values.

The QSPR models developed for patiromer-drug binding *in vitro* reflect interactions between multiple drug properties, rather than a univariate relationship to lipophilicity or charge (in contrast, lipophilicity [LogD] was identified as the major determinant of *in vitro* binding to colesevelam [5]). For patiromer, ionization potential and availability of surface hydrogen bond acceptors were key factors over the physiological pH range of the gut. At the lowest pH tested (3.0), lipophilicity (QPlogPw) was also influential, but this factor was not predictive in matrices with higher pH values.

When test drugs were stratified based on the extent of binding to patiromer (strong, weak, or none), shared molecular characteristics emerged for each set. In general, non-binding test drugs had multiple polar functional groups. They included examples from multiple drug classes (eg, amoxicillin, glipizide, furosemide), and relatively small, highly polar compounds, such as allopurinol and warfarin. In particular, the inclusion of carboxylates and multiple hydroxyl groups appears to greatly decrease the likelihood of binding to patiromer.

Those drugs that did bind patiromer *in vitro* tended to have fewer polar groups and were more lipophilic than the nonbinders. The weak binders were generally neutral to weakly basic, with limited polar functionality, as exemplified by clopidogrel or metoprolol. The strongest *in vitro* binding to patiromer is predicted to occur with drugs that contain basic amines that are not balanced by a polar functionality, eg, a carboxylate or cyano group or multiple amides, in close proximity to the basic group.

It is important to note, however, that while the QSPR models for patiromer have predictive value for *in vitro* binding, additional factors, such as drug dissolution, absorption and binding to other gut contents, intervene *in vivo* to determine whether a significant DDI will occur. Our findings (4) and those of Walker *et al*. (5) both suggest that *in vitro* polymer-drug binding studies tend to over-predict clinically meaningful DDIs. In the case of patiromer, only 3 of 12 drugs that bound patiromer *in vitro* showed a potentially clinically relevant pharmacokinetics (PK) change, defined as 80–125% of the 90% confidence interval for the area under the plasma concentration–time curve from 0 to infinity (AUC). No clinically relevant DDIs were observed when test drug and patiromer were given 3 h apart (4). Of the four strong *in vitro* patiromer binders that were tested clinically, only ciprofloxacin showed a decrease in systemic absorption when administered together with patiromer. Because both ciprofloxacin and levothyroxine bind calcium, the effects on PK observed upon administration of either drug together with patiromer may reflect binding to patiromer’s calcium counter-ion, rather than to the anion itself.

The data generated for metformin are also illustrative of the principle that other factors beyond *in vitro* binding influence the potential for clinical DDIs with nonabsorbable polymeric drugs. Metformin is a weak binder *in vitro*, but also showed a moderate reduction in AUC in healthy volunteers when co-administered with patiromer. Metformin has low permeability and its uptake is transporter-mediated, thus saturable (15). Interaction with patiromer may compete with transporter-mediated absorption and, in the presence of patiromer, a greater proportion of the drug may fail to bind to transporters during the gut transit period, leading to a reduced AUC.

## Conclusion

The potential for polymeric therapeutics to reduce the bioavailability of co-administered drugs is limited to binding that may occur in the lumen of the gastrointestinal tract, because the polymers are not systemically absorbed. In this paper we demonstrate that an in silico QSPR approach can be used to characterize the drivers of *in vitro* binding and has the potential to predict which drugs are more likely to show a binding interaction. Thus, *in vitro* binding combined with QSPR can be used proactively to narrow the subset of drugs that are tested clinically for drug interactions with polymers.

## Electronic supplementary material

ESM 1 (DOCX 175 kb)

## Abbreviations

AB: Acetate buffer
AUC: Area under the curve
BCS: Biopharmaceutics Classification System
CI: Confidence interval
Conc: Concentration
CV: Cross-validation
DDI: Drug-drug interaction
HCl: Hydrochloride
IP.ev,PM3: Calculated ionization potential
LogD: Lipophilicity
LogP: Partition coefficient
PK: Pharmacokinetics
QPlogPw,QikProp: Predicted water gas partition coefficient
QSPR: Quantitative structure-property relationship
SAA1: Sum of surface area on acceptor atoms
SGF: Simulated gastric fluid
SIF: Simulated intestinal fluid
X.amide: Number of non-conjugated amide groups
X.noncon: Number of ring atoms not able to form conjugated aromatic systems
