# Supplementary material for: Use of QSPR Modeling to Characterize In Vitro Binding of Drugs to a Gut-Restricted Polymer
Source: Pharm Res. 2018 Mar 8;35(4):89. doi: 10.1007/s11095-018-2356-y (PMC5843698; doi:10.1007/s11095-018-2356-y)
Supplement: Supplementary file 1 — (DOCX 175 kb) [file 11095_2018_2356_MOESM1_ESM.docx]

**Supplementary Appendix**

This appendix has been provided by the authors to give readers additional information about their work.

Supplement to: Brew CT, Blake JF, Mistry WW, et al. Use of QSPR modeling to characterize in vitro binding of drugs to a gut-restricted polymer

Table of Contents

[Supplementary Figure S1. Concentration independence (Ci) of in vitro drug binding to patiromer at physiologically relevant concentrations 2](#_Toc504735157)

[Supplementary Table SI. Complete set of physicochemical parameters used in QSPR. 3](#_Toc504735158)

[Supplementary Table SII. QSPR input data and variables 14](#_Toc504735159)

# Supplementary Figure S1. Concentration independence (Ci) of *in vitro* drug binding to patiromer at physiologically relevant concentrations

**
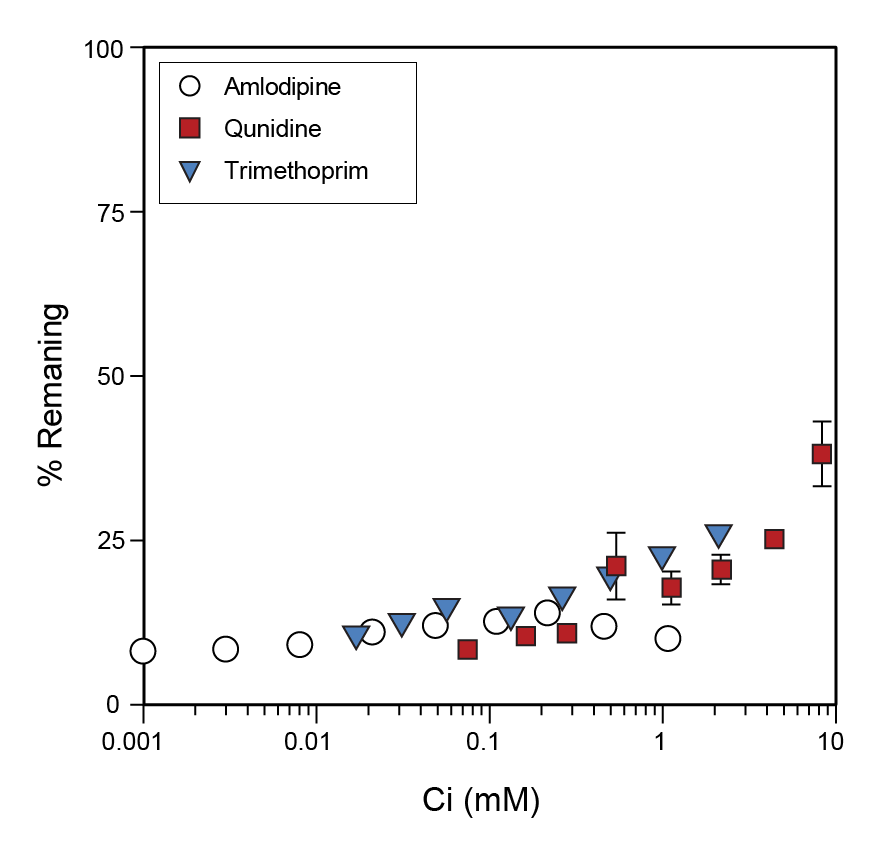
**

# Supplementary Table SI. Complete set of physicochemical parameters used in QSPR.

| Drug | SGF | AB | | SIF | | Number of rotatable bonds | | dipole | | SASA | | FOSA | | FISA | PISA | | WPSA | | volume | | donorHB | |  |
| --- | --- | --- | --- | --- | --- | --- | --- | --- | --- | --- | --- | --- | --- | --- | --- | --- | --- | --- | --- | --- | --- | --- | --- |
| Allopurinol | 85.7 | 90.1 | | 94.2 | | 0 | | 6.028 | | 296.867 | | 0 | | 186.312 | 110.555 | | 0 | | 443.562 | | 2 | |  |
| Amlodipine | 10.8 | 36.6 | | 13.1 | | 10 | | 3.299 | | 706.529 | | 404.216 | | 135.948 | 143.617 | | 22.747 | | 1269.755 | | 2 | |  |
| Amoxicillin |  | 99.2 | | 99.4 | | 4 | | 4.562 | | 542.67 | | 145.923 | | 273.326 | 109.524 | | 13.896 | | 1017.979 | | 4.25 | |  |
| Apixaban | 75.6 | 97.3 | | 97.8 | | 5 | | 7.954 | | 777.264 | | 346.444 | | 184.187 | 246.633 | | 0 | | 1401.959 | | 2 | |  |
| Aspirin | 100.7 | 99.8 | | 99.9 | | 3 | | 2.218 | | 381.085 | | 90.726 | | 130.258 | 160.102 | | 0 | | 610.435 | | 1 | |  |
| Atorvastatin | 91.2 | 93.3 | | 101.1 | | 12 | | 7.349 | | 914.053 | | 231.138 | | 185.596 | 450.299 | | 47.019 | | 1730.692 | | 3 | |  |
| Cephalexin | 88.7 | 95 | | 104.4 | | 4 | | 7.299 | | 571.497 | | 168.462 | | 221.529 | 172.761 | | 8.744 | | 1018.975 | | 3.25 | |  |
| Cinacalcet | 13.4 | 19.3 | | 18.3 | | 7 | | 2.724 | | 675.319 | | 163.174 | | 8.608 | 386.887 | | 116.65 | | 1174.386 | | 1 | |  |
| Ciprofloxacin | 18 | 24.8 | | 6.9 | | 3 | | 8.521 | | 575.132 | | 295.534 | | 176.809 | 70.617 | | 32.172 | | 1011.241 | | 1 | |  |
| Clopidogrel | 66 |  | |  | | 4 | | 5.433 | | 545.167 | | 176.115 | | 43.442 | 240.303 | | 85.307 | | 960.929 | | 0 | |  |
| Digoxin |  | 109.3 | | 103.4 | | 7 | | 14.181 | | 1119.925 | | 830.274 | | 266.405 | 23.247 | | 0 | | 2216.223 | | 6 | |  |
| Furosemide | 67.3 | 94.5 | | 79.1 | | 5 | | 7.327 | | 533.796 | | 35.747 | | 232.829 | 211.471 | | 53.75 | | 901.877 | | 3 | |  |
| Glipizide | 72.9 | 96.5 | | 98.5 | | 7 | | 13.067 | | 814.621 | | 395.493 | | 217.06 | 200.395 | | 1.672 | | 1426.638 | | 2 | |  |
| Lisinopril | 77.9 | 100.7 | | 98.5 | | 12 | | 7.106 | | 739.945 | | 277.343 | | 271.887 | 190.715 | | 0 | | 1333.129 | | 5 | |  |
| Metformin | 48.9 | 81.8 | | 80.2 | | 3 | | 8.719 | | 343.137 | | 161.551 | | 181.586 | 0 | | 0 | | 518.97 | | 5 | |  |
| Metoprolol | 71.7 | 85.9 | | 68.6 | | 9 | | 3.007 | | 609.847 | | 419.043 | | 54.243 | 136.561 | | 0 | | 1024.89 | | 2 | |  |
| Phenytoin | 83.4 | 90.2 | | 92.8 | | 2 | | 4.005 | | 450.039 | | 0 | | 110.583 | 339.456 | | 0 | | 781.174 | | 2 | |  |
| Quinidine | 12.6 | 43.1 | | 24.3 | | 4 | | 3.621 | | 577.821 | | 328.613 | | 60.617 | 188.591 | | 0 | | 1048.635 | | 1 | |  |
| Riboflavin | 95.6 |  | | 96.5 | | 5 | | 14.639 | | 607.463 | | 255.167 | | 283.673 | 68.623 | | 0 | | 1087.92 | | 5 | |  |
| Rivaroxaban | 71.9 | 92.9 | | 95.1 | | 5 | | 3.825 | | 692.575 | | 230.92 | | 145.763 | 206.763 | | 109.129 | | 1227.414 | | 1 | |  |
| Spironolactone | 78.7 | 98.6 | | 96.8 | | 2 | | 1.875 | | 663.796 | | 469.108 | | 158.051 | 23.425 | | 13.212 | | 1258.564 | | 0 | |  |
| Trimethoprim | 36.3 | 55.3 | | 28.4 | | 5 | | 4.19 | | 519.718 | | 280.753 | | 148.285 | 90.68 | | 0 | | 918.078 | | 4 | |  |
| Valsartan | 86.4 | 101.4 | | 98 | | 10 | | 5.516 | | 766.682 | | 327.696 | | 199.795 | 239.192 | | 0 | | 1406.704 | | 2 | |  |
| Verapamil | 51.7 | 88.6 | | 77.9 | | 14 | | 5.452 | | 847.646 | | 632.427 | | 55.494 | 159.724 | | 0 | | 1559.877 | | 0 | |  |
| Warfarin | 66.3 | 92.5 | | 97.3 | | 4 | | 5.502 | | 548.176 | | 98.503 | | 109.739 | 339.933 | | 0 | | 976.819 | | 0 | |  |
| Drug | accptHB | | dip^2/V | | ACxDN^.5/SA | | glob | | QPpolrz | | QPlogPC16 | | QPlogPoct | | | QPlogPw | | QPlogPo/w | | QPlogS | | CIQPlogS | |
| Allopurinol | 4.5 | | 0.081908 | | 0.021437 | | 0.947486 | | 12.653 | | 5.096 | | 10.465 | | | 9.814 | | -0.55 | | -2.258 | | -1.151 | |
| Amlodipine | 6.2 | | 0.00857 | | 0.01241 | | 0.80263 | | 40.896 | | 12.634 | | 19.664 | | | 10.601 | | 3.01 | | -4.374 | | -4.601 | |
| Amoxicillin | 8 | | 0.020447 | | 0.030391 | | 0.901825 | | 31.779 | | 11.639 | | 21.798 | | | 21.458 | | -2.714 | | 0.031 | | -1.304 | |
| Apixaban | 10.25 | | 0.045123 | | 0.01865 | | 0.779388 | | 51.024 | | 14.87 | | 26.326 | | | 17.104 | | 2.691 | | -6.315 | | -5.669 | |
| Aspirin | 4.5 | | 0.008059 | | 0.011808 | | 0.913206 | | 18.52 | | 6.275 | | 9.73 | | | 8.067 | | 1.189 | | -1.716 | | -1.578 | |
| Atorvastatin | 6.9 | | 0.031203 | | 0.013075 | | 0.762676 | | 59.703 | | 19.378 | | 29.086 | | | 14.804 | | 6.844 | | -8.279 | | -9.022 | |
| Cephalexin | 7.25 | | 0.052288 | | 0.02287 | | 0.856894 | | 33.065 | | 11.287 | | 20.538 | | | 16.599 | | -1.474 | | -1.726 | | -2.057 | |
| Cinacalcet | 1.5 | | 0.00632 | | 0.002221 | | 0.797131 | | 40.687 | | 11.404 | | 15.813 | | | 5.694 | | 6.336 | | -6.362 | | -5.775 | |
| Ciprofloxacin | 6 | | 0.071802 | | 0.010432 | | 0.847163 | | 34.348 | | 9.367 | | 17.136 | | | 9.952 | | 0.28 | | -3.794 | | -3.022 | |
| Clopidogrel | 4 | | 0.030718 | | 0 | | 0.863833 | | 32.671 | | 9.78 | | 13.302 | | | 6.269 | | 3.395 | | -3.118 | | -3.709 | |
| Digoxin | 22.45 | | 0.090734 | | 0.049102 | | 0.734037 | | 75.056 | | 22.578 | | 47.534 | | | 32.638 | | 1.383 | | -6.23 | | -5.996 | |
| Furosemide | 7 | | 0.059529 | | 0.022713 | | 0.845709 | | 28.106 | | 10.718 | | 18.646 | | | 14.01 | | 1.861 | | -4.418 | | -3.791 | |
| Glipizide | 8 | | 0.119685 | | 0.013888 | | 0.752348 | | 48.359 | | 15.194 | | 24.997 | | | 15.084 | | 2.836 | | -6.337 | | -5.084 | |
| Lisinopril | 9.5 | | 0.037877 | | 0.028708 | | 0.791676 | | 40.672 | | 15.074 | | 26.572 | | | 20.147 | | -1.195 | | -1.481 | | -1.196 | |
| Metformin | 3.5 | | 0.146501 | | 0.022808 | | 0.910176 | | 13.331 | | 5.392 | | 15.074 | | | 12.109 | | -0.768 | | -0.833 | | -0.649 | |
| Metoprolol | 5.65 | | 0.008822 | | 0.013102 | | 0.806113 | | 29.745 | | 9.462 | | 14.821 | | | 8.773 | | 1.888 | | -1.425 | | -1.763 | |
| Phenytoin | 3.5 | | 0.020536 | | 0.010998 | | 0.911478 | | 28.35 | | 9.345 | | 14.543 | | | 10.24 | | 2.469 | | -3.846 | | -3.83 | |
| Quinidine | 5.45 | | 0.012505 | | 0.009432 | | 0.863882 | | 34.403 | | 9.992 | | 15.434 | | | 8.535 | | 3.418 | | -3.269 | | -3.218 | |
| Riboflavin | 12.3 | | 0.196994 | | 0.045276 | | 0.842125 | | 32.261 | | 11.944 | | 27.443 | | | 21.03 | | -0.971 | | -2.341 | | -2.963 | |
| Rivaroxaban | 10.7 | | 0.011918 | | 0.01545 | | 0.800495 | | 43.015 | | 13.133 | | 22.152 | | | 15.164 | | 2.127 | | -4.869 | | -4.758 | |
| Spironolactone | 7 | | 0.002792 | | 0 | | 0.849273 | | 43.153 | | 11.272 | | 18.155 | | | 9.009 | | 3.081 | | -4.863 | | -4.895 | |
| Trimethoprim | 5.25 | | 0.01912 | | 0.020203 | | 0.878989 | | 26.959 | | 9.429 | | 17.388 | | | 12.081 | | 0.916 | | -2.812 | | -3.586 | |
| Valsartan | 8.5 | | 0.021632 | | 0.015679 | | 0.791926 | | 46.006 | | 14.911 | | 23.162 | | | 15.14 | | 3.216 | | -4.763 | | -5.023 | |
| Verapamil | 6.5 | | 0.019057 | | 0 | | 0.767381 | | 48.808 | | 14.443 | | 19.07 | | | 6.537 | | 3.799 | | -5.598 | | -6.384 | |
| Warfarin | 6.5 | | 0.030985 | | 0 | | 0.868536 | | 33.616 | | 10.519 | | 15.039 | | | 9.617 | | 2.152 | | -2.65 | | -3.5 | |

| Drug | QPlogHERG | QPPCaco | QPlogBB | QPPMDCK | QPlogKp | IP(eV) | EA(eV) | QPlogKhsa | SAfluorine | SAamideO | PSA | #NandO |
| --- | --- | --- | --- | --- | --- | --- | --- | --- | --- | --- | --- | --- |
| Allopurinol | -2.909 | 169.476 | -0.82 | 72.628 | -4.564 | 9.547 | 0.775 | -0.738 | 0 | 0 | 88.237 | 5 |
| Amlodipine | -6.137 | 126.941 | -0.871 | 78.332 | -4.814 | 8.425 | 0.423 | 0.475 | 0 | 0 | 106.092 | 7 |
| Amoxicillin | 0.187 | 0.459 | -1.606 | 0.789 | -7.658 | 9.232 | 0.587 | -1.093 | 0 | 68.14 | 158.849 | 8 |
| Apixaban | -6.278 | 177.525 | -1.579 | 76.364 | -3.853 | 8.856 | 0.552 | 0.301 | 0 | 0 | 132.093 | 9 |
| Aspirin | -1.892 | 145.97 | -0.598 | 78.606 | -3.164 | 9.868 | 0.621 | -0.732 | 0 | 0 | 83.214 | 4 |
| Atorvastatin | -5.712 | 43.601 | -2.172 | 38.531 | -2.202 | 8.591 | 0.377 | 1.138 | 47.019 | 0 | 119.816 | 7 |
| Cephalexin | -2.216 | 3.007 | -1.329 | 2.511 | -6.576 | 9.421 | 0.875 | -0.667 | 0 | 27.316 | 134.587 | 7 |
| Cinacalcet | -7.386 | 2047.233 | 0.796 | 5170.991 | -1.802 | 8.605 | 0.787 | 1.283 | 116.65 | 0 | 12.225 | 1 |
| Ciprofloxacin | -3.26 | 13.174 | -0.662 | 9.695 | -6.496 | 8.742 | 0.821 | 0.013 | 32.172 | 0 | 98.355 | 6 |
| Clopidogrel | -5.607 | 956.844 | 0.602 | 1530.505 | -3.248 | 9.058 | 0.389 | 0.21 | 0 | 0 | 35.36 | 3 |
| Digoxin | -5.946 | 29.484 | -3.456 | 10.969 | -5.195 | 10.258 | 0.428 | -0.442 | 0 | 0 | 201.255 | 14 |
| Furosemide | -3.23 | 15.545 | -1.837 | 13.758 | -4.49 | 8.944 | 0.871 | -0.621 | 0 | 0 | 130.05 | 7 |
| Glipizide | -5.121 | 62.774 | -2.411 | 35.9 | -4.142 | 9.798 | 1.073 | 0.233 | 0 | 17.555 | 149.713 | 9 |
| Lisinopril | -2.275 | 0.069 | -2.315 | 0.049 | -8.736 | 9.398 | -0.046 | -0.928 | 0 | 22.807 | 159.406 | 8 |
| Metformin | -2.985 | 187.898 | -1.018 | 81.198 | -8.799 | 8.573 | -0.589 | -0.831 | 0 | 0 | 102.212 | 5 |
| Metoprolol | -6.047 | 755.81 | -0.197 | 404.397 | -3.141 | 8.951 | -0.256 | -0.144 | 0 | 0 | 50.323 | 4 |
| Phenytoin | -4.583 | 885.606 | -0.324 | 433.834 | -2.362 | 9.836 | 0.289 | 0.057 | 0 | 0 | 78.329 | 4 |
| Quinidine | -5.36 | 657.618 | 0.119 | 347.921 | -3.555 | 8.58 | 0.756 | 0.216 | 0 | 0 | 45.785 | 4 |
| Riboflavin | -4.414 | 20.222 | -2.646 | 7.297 | -5.642 | 8.745 | 1.39 | -0.856 | 0 | 0 | 174.023 | 10 |
| Rivaroxaban | -5.746 | 410.796 | -0.866 | 749.089 | -3.19 | 8.847 | 1.224 | -0.323 | 0 | 0 | 116.924 | 8 |
| Spironolactone | -4.083 | 314.124 | -0.986 | 167.167 | -4.158 | 9.923 | 0.553 | 0.314 | 0 | 0 | 97.222 | 4 |
| Trimethoprim | -3.923 | 388.792 | -1.138 | 178.19 | -3.261 | 8.362 | -0.072 | -0.281 | 0 | 0 | 93.693 | 7 |
| Valsartan | -2.858 | 22.087 | -2.163 | 15.23 | -3.399 | 9.236 | 0.666 | -0.169 | 0 | 20.191 | 129.341 | 8 |
| Verapamil | -6.784 | 735.441 | -0.51 | 392.63 | -2.698 | 8.754 | -0.028 | 0.813 | 0 | 0 | 57.544 | 6 |
| Warfarin | -5.28 | 902.073 | -0.647 | 442.56 | -1.96 | 9.564 | 1.065 | -0.455 | 0 | 0 | 85.107 | 4 |

| Drug | #ringatoms | #in56 | #noncon | #nonHatm | Jm | AlogP | Molecular weight | Hydrogen bond acceptors | Hydrogen bond donors | Polar SA | HYDROPHOBIC |
| --- | --- | --- | --- | --- | --- | --- | --- | --- | --- | --- | --- |
| Allopurinol | 9 | 9 | 0 | 10 | 0.554 | -0.837 | 136.1138 | 3 | 2 | 74.43 | 0 |
| Amlodipine | 12 | 12 | 1 | 28 | 0 | 1.5765 | 408.8857 | 5 | 1 | 99.88 | 381.633 |
| Amoxicillin | 13 | 9 | 4 | 25 | 0.01 | -0.1105 | 365.4109 | 4 | 2 | 158.26 | 159.26 |
| Apixaban | 27 | 27 | 6 | 34 | 0 | 2.5005 | 459.5091 | 5 | 1 | 110.76 | 354.821 |
| Aspirin | 6 | 6 | 0 | 13 | 3.596 | 1.2021 | 180.1617 | 2 | 0 | 63.6 | 102.38 |
| Atorvastatin | 23 | 23 | 0 | 41 | 0 | 5.4402 | 558.6557 | 3 | 3 | 111.79 | 230.569 |
| Cephalexin | 14 | 10 | 3 | 24 | 0.001 | 0.1989 | 347.3956 | 3 | 1 | 138.03 | 176.486 |
| Cinacalcet | 16 | 16 | 0 | 26 | 0.002 | 5.9568 | 357.4225 | 0 | 0 | 12.03 | 157.078 |
| Ciprofloxacin | 19 | 16 | 7 | 24 | 0 | 3.0121 | 331.3497 | 1 | 0 | 74.57 | 299.546 |
| Clopidogrel | 15 | 15 | 3 | 21 | 0.139 | 3.5082 | 321.8284 | 2 | 0 | 57.78 | 182.419 |
| Digoxin | 40 | 40 | 33 | 55 | 0 | 1.8927 | 780.9588 | 14 | 6 | 203.06 | 791.884 |
| Furosemide | 11 | 11 | 0 | 21 | 0.011 | 1.1191 | 330.7489 | 2 | 2 | 131.01 | 45.925 |
| Glipizide | 18 | 18 | 6 | 31 | 0 | 2.0966 | 445.5444 | 5 | 3 | 138.53 | 392.686 |
| Lisinopril | 11 | 11 | 4 | 29 | 0 | 1.4598 | 405.4983 | 1 | 0 | 132.96 | 274.322 |
| Metformin | 0 | 0 | 0 | 9 | 0 | -0.7411 | 129.1658 | 2 | 2 | 88.99 | 186.28 |
| Metoprolol | 6 | 6 | 0 | 19 | 0.649 | 1.7572 | 267.3714 | 3 | 1 | 50.72 | 394.818 |
| Phenytoin | 17 | 17 | 1 | 19 | 0.678 | 2.2996 | 252.2751 | 2 | 2 | 58.2 | 0 |
| Quinidine | 18 | 18 | 7 | 24 | 0.137 | 2.7338 | 324.4265 | 3 | 1 | 45.59 | 261.587 |
| Riboflavin | 14 | 14 | 0 | 27 | 0.004 | 0.9794 | 376.3722 | 8 | 5 | 161.56 | 240.938 |
| Rivaroxaban | 22 | 22 | 5 | 29 | 0.004 | 2.6071 | 435.8894 | 5 | 1 | 116.42 | 228.946 |
| Spironolactone | 21 | 21 | 16 | 29 | 0 | 3.4852 | 416.5842 | 5 | 0 | 85.74 | 465.195 |
| Trimethoprim | 12 | 12 | 0 | 21 | 0.409 | 1.5463 | 290.3246 | 4 | 2 | 105.51 | 265.561 |
| Valsartan | 17 | 17 | 0 | 32 | 0.003 | 4.7109 | 435.5304 | 4 | 1 | 112.07 | 325.783 |
| Verapamil | 12 | 12 | 0 | 33 | 0 | 5.535 | 454.6149 | 5 | 0 | 63.95 | 612.106 |
| Warfarin | 16 | 16 | 1 | 23 | 9.506 | 2.6942 | 308.337 | 4 | 0 | 60.44 | 122.44 |

| Drug | POLAR | PITYPE | WEAKPOLAR | | TOTALAREA | TOTALVOLUME | NAA | NDH | SAAA1 | SAAA2 | SAAA3 | SADH1 | SADH2 |
| --- | --- | --- | --- | --- | --- | --- | --- | --- | --- | --- | --- | --- | --- |
| Allopurinol | 179.621 | 125.72 | 0 | 305.341 | | 456.838 | 3 | 2 | 107.292 | 35.764 | 0.351 | 54.028 | 27.014 |
| Amlodipine | 145.048 | 145.025 | 23.456 | 695.162 | | 1259.502 | 6 | 3 | 77.982 | 12.997 | 0.112 | 62.651 | 20.884 |
| Amoxicillin | 243.947 | 101.204 | 13.741 | 518.151 | | 1002.096 | 6 | 5 | 190.044 | 31.674 | 0.367 | 51.125 | 10.225 |
| Apixaban | 187.918 | 248.279 | 0 | 791.018 | | 1410.584 | 5 | 2 | 131.016 | 26.203 | 0.166 | 39.238 | 19.619 |
| Aspirin | 101.381 | 161.266 | 0 | 365.027 | | 596.593 | 4 | 1 | 101.381 | 25.345 | 0.278 | 0 | 0 |
| Atorvastatin | 166.485 | 444.587 | 46.997 | 888.637 | | 1709.923 | 5 | 4 | 134.833 | 26.967 | 0.152 | 30.195 | 7.549 |
| Cephalexin | 186.6 | 174.426 | 9.412 | 546.924 | | 998.966 | 5 | 4 | 153.7 | 30.74 | 0.281 | 30.283 | 7.571 |
| Cinacalcet | 11.907 | 387.595 | 116.59 | 673.17 | | 1168.29 | 1 | 1 | 4.875 | 4.875 | 0.007 | 7.032 | 7.032 |
| Ciprofloxacin | 133.234 | 86.045 | 32.616 | 551.44 | | 986.024 | 4 | 2 | 108.283 | 27.071 | 0.196 | 23.683 | 11.841 |
| Clopidogrel | 36.133 | 243.658 | 87.737 | 549.947 | | 958.661 | 3 | 0 | 36.133 | 12.044 | 0.066 | 0 | 0 |
| Digoxin | 309.171 | 38.705 | 0 | 1139.76 | | 2229.682 | 14 | 6 | 225.097 | 16.078 | 0.197 | 84.074 | 14.012 |
| Furosemide | 214.893 | 209.588 | 53.239 | 523.644 | | 903.573 | 5 | 4 | 144.229 | 28.846 | 0.275 | 51.875 | 12.969 |
| Glipizide | 201.161 | 199.943 | 1.67 | 795.46 | | 1409.407 | 6 | 3 | 155.062 | 25.844 | 0.195 | 35.173 | 11.724 |
| Lisinopril | 230.519 | 191.616 | 0 | 696.457 | | 1301.08 | 7 | 5 | 170.919 | 24.417 | 0.245 | 59.235 | 11.847 |
| Metformin | 158.431 | 0 | 0 | 344.712 | | 525.516 | 2 | 4 | 73.008 | 36.504 | 0.212 | 60.127 | 15.032 |
| Metoprolol | 83.516 | 137.416 | 0 | 615.75 | | 1031.685 | 4 | 2 | 54.311 | 13.578 | 0.088 | 29.205 | 14.603 |
| Phenytoin | 125.421 | 352.946 | 0 | 478.367 | | 813.128 | 2 | 2 | 81.047 | 40.524 | 0.169 | 36.181 | 18.091 |
| Quinidine | 62.976 | 243.008 | 0 | 567.571 | | 1039.908 | 4 | 1 | 62.976 | 15.744 | 0.111 | 0 | 0 |
| Riboflavin | 293.821 | 100.689 | 0 | 635.448 | | 1118.599 | 8 | 5 | 199.937 | 24.992 | 0.315 | 86.308 | 17.262 |
| Rivaroxaban | 157.643 | 223.195 | 107.101 | 716.885 | | 1249.953 | 5 | 1 | 145.245 | 29.049 | 0.203 | 8.264 | 8.264 |
| Spironolactone | 134.068 | 43.815 | 13.976 | 657.054 | | 1234.472 | 4 | 0 | 134.068 | 33.517 | 0.204 | 0 | 0 |
| Trimethoprim | 194.877 | 85.972 | 0 | 546.411 | | 953.726 | 5 | 4 | 70.247 | 14.049 | 0.129 | 88.91 | 22.228 |
| Valsartan | 173.773 | 235.996 | 0 | 735.552 | | 1382.7 | 6 | 2 | 131.855 | 21.976 | 0.179 | 31.2 | 15.6 |
| Verapamil | 96.776 | 151.893 | 0 | 860.775 | | 1563.737 | 6 | 0 | 96.776 | 16.129 | 0.112 | 0 | 0 |
| Warfarin | 102.664 | 340.29 | 0 | 565.394 | | 990.219 | 4 | 0 | 102.664 | 25.666 | 0.182 | 0 | 0 |

| Drug | SADH3 | OVALITY | GRAVI | POLARIZABILITY | MOSTNEGATIVECHARGE | MOSTPOSITIVECHARGE | SUMALLCHARGES | PPSA1 | PPSA2 |
| --- | --- | --- | --- | --- | --- | --- | --- | --- | --- |
| Allopurinol | 0.177 | 1.064 | 1040.342 | 13.084 | -0.457 | 0.482 | 3.795 | 174.163 | 330.493 |
| Amlodipine | 0.09 | 1.233 | 2656.406 | 42.804 | -2.42 | 0.826 | 43.574 | 466.961 | 10173.62 |
| Amoxicillin | 0.099 | 1.07 | 2464.581 | 36.385 | -2.499 | 0.833 | 35.08 | 266.537 | 4675.016 |
| Apixaban | 0.05 | 1.3 | 3320.595 | 50.82 | -2.295 | 0.84 | 44.197 | 482.034 | 10652.29 |
| Aspirin | 0 | 1.065 | 1212.826 | 18.333 | -2.411 | 0.803 | 15.714 | 171.838 | 1350.113 |
| Atorvastatin | 0.034 | 1.285 | 3764.502 | 63.814 | -2.505 | 0.83 | 59.383 | 504.552 | 14980.85 |
| Cephalexin | 0.055 | 1.132 | 2366.837 | 35.073 | -2.415 | 0.832 | 31.128 | 300.762 | 4681.097 |
| Cinacalcet | 0.01 | 1.255 | 2373.237 | 40.654 | -2.5 | 0.821 | 36.408 | 355.075 | 6463.837 |
| Ciprofloxacin | 0.043 | 1.151 | 2338.836 | 35.169 | -1.594 | 0.813 | 31.43 | 323.97 | 5091.262 |
| Clopidogrel | 0 | 1.17 | 2087.483 | 32.577 | -2.255 | 0.801 | 26.588 | 293.921 | 3907.335 |
| Digoxin | 0.074 | 1.381 | 5260.25 | 87.103 | -2.602 | 0.802 | 104.667 | 798.859 | 41807.07 |
| Furosemide | 0.099 | 1.159 | 2406.374 | 28.873 | -1.756 | 1.344 | 23.215 | 202.813 | 2354.16 |
| Glipizide | 0.044 | 1.309 | 3275.091 | 46.832 | -2.388 | 1.338 | 49.031 | 490.572 | 12026.61 |
| Lisinopril | 0.085 | 1.208 | 2641.775 | 45.409 | -1.764 | 0.824 | 52.6 | 415.126 | 10917.71 |
| Metformin | 0.174 | 1.095 | 839.307 | 13.971 | -2.403 | 0.842 | 20.342 | 215.844 | 2195.4 |
| Metoprolol | 0.047 | 1.247 | 1710.548 | 31.995 | -2.543 | 0.812 | 40.149 | 439.988 | 8832.629 |
| Phenytoin | 0.076 | 1.135 | 1799.996 | 28.44 | -1.049 | 0.834 | 21.917 | 240.673 | 2637.442 |
| Quinidine | 0 | 1.143 | 2235.604 | 38.98 | -2.294 | 0.799 | 38.667 | 358.974 | 6940.187 |
| Riboflavin | 0.136 | 1.219 | 2598.125 | 38.713 | -2.441 | 0.833 | 35.971 | 350.541 | 6304.608 |
| Rivaroxaban | 0.012 | 1.278 | 2997.983 | 41.643 | -1.601 | 0.827 | 32.06 | 338.938 | 5433.198 |
| Spironolactone | 0 | 1.181 | 2742.476 | 47.015 | -2.563 | 0.806 | 57.09 | 425.524 | 12146.63 |
| Trimethoprim | 0.163 | 1.166 | 2040.44 | 31.813 | -2.311 | 0.84 | 32.464 | 346.945 | 5631.68 |
| Valsartan | 0.042 | 1.225 | 2975.479 | 49.778 | -2.521 | 0.835 | 48.979 | 446.014 | 10922.68 |
| Verapamil | 0 | 1.321 | 3010.356 | 54.19 | -2.507 | 0.801 | 62.93 | 578.849 | 18213.58 |
| Warfarin | 0 | 1.177 | 2108.666 | 34.687 | -2.488 | 0.803 | 29.26 | 299.143 | 4376.407 |

| Drug | PPSA3 | PNSA1 | PNSA2 | PNSA3 | FPSA1 | FPSA2 | FPSA3 | FNSA1 | FNSA2 | FNSA3 | WPSA1 | WPSA2 |
| --- | --- | --- | --- | --- | --- | --- | --- | --- | --- | --- | --- | --- |
| Allopurinol | 42.128 | 131.178 | -248.927 | -48.243 | 0.57 | 1.082 | 0.138 | 0.43 | -0.815 | -0.158 | 53.179 | 100.913 |
| Amlodipine | 369.127 | 228.201 | -4971.78 | -263.262 | 0.672 | 14.635 | 0.531 | 0.328 | -7.152 | -0.379 | 324.613 | 7072.31 |
| Amoxicillin | 204.845 | 251.614 | -4413.28 | -235.302 | 0.514 | 9.022 | 0.395 | 0.486 | -8.517 | -0.454 | 138.106 | 2422.365 |
| Apixaban | 372.043 | 308.984 | -6828.14 | -258.29 | 0.609 | 13.467 | 0.47 | 0.391 | -8.632 | -0.327 | 381.297 | 8426.154 |
| Aspirin | 131.679 | 193.188 | -1517.85 | -159.889 | 0.471 | 3.699 | 0.361 | 0.529 | -4.158 | -0.438 | 62.726 | 492.827 |
| Atorvastatin | 386.328 | 384.086 | -11404.1 | -326.661 | 0.568 | 16.858 | 0.435 | 0.432 | -12.833 | -0.368 | 448.363 | 13312.55 |
| Cephalexin | 231.459 | 246.162 | -3831.3 | -217.177 | 0.55 | 8.559 | 0.423 | 0.45 | -7.005 | -0.397 | 164.494 | 2560.202 |
| Cinacalcet | 272.406 | 318.095 | -5790.65 | -208.265 | 0.527 | 9.602 | 0.405 | 0.473 | -8.602 | -0.309 | 239.026 | 4351.26 |
| Ciprofloxacin | 238.907 | 227.47 | -3574.74 | -174.64 | 0.587 | 9.233 | 0.433 | 0.413 | -6.483 | -0.317 | 178.65 | 2807.528 |
| Clopidogrel | 225.159 | 256.026 | -3403.58 | -165.424 | 0.534 | 7.105 | 0.409 | 0.466 | -6.189 | -0.301 | 161.641 | 2148.827 |
| Digoxin | 625.234 | 340.901 | -17840.6 | -408.886 | 0.701 | 36.681 | 0.549 | 0.299 | -15.653 | -0.359 | 910.507 | 47650.02 |
| Furosemide | 151.902 | 320.831 | -3724.05 | -206.966 | 0.387 | 4.496 | 0.29 | 0.613 | -7.112 | -0.395 | 106.202 | 1232.741 |
| Glipizide | 381.644 | 304.888 | -7474.47 | -247.841 | 0.617 | 15.119 | 0.48 | 0.383 | -9.396 | -0.312 | 390.23 | 9566.68 |
| Lisinopril | 324.859 | 281.332 | -7398.95 | -253.707 | 0.596 | 15.676 | 0.466 | 0.404 | -10.624 | -0.364 | 289.117 | 7603.717 |
| Metformin | 169.634 | 128.867 | -1310.74 | -209.954 | 0.626 | 6.369 | 0.492 | 0.374 | -3.802 | -0.609 | 74.404 | 756.78 |
| Metoprolol | 341.219 | 175.763 | -3528.38 | -232.695 | 0.715 | 14.345 | 0.554 | 0.285 | -5.73 | -0.378 | 270.923 | 5438.693 |
| Phenytoin | 186.595 | 237.694 | -2604.79 | -167.465 | 0.503 | 5.513 | 0.39 | 0.497 | -5.445 | -0.35 | 115.13 | 1261.666 |
| Quinidine | 271.631 | 208.597 | -4032.9 | -225.96 | 0.632 | 12.228 | 0.479 | 0.368 | -7.106 | -0.398 | 203.743 | 3939.047 |
| Riboflavin | 260.61 | 284.907 | -5124.16 | -257.506 | 0.552 | 9.922 | 0.41 | 0.448 | -8.064 | -0.405 | 222.751 | 4006.249 |
| Rivaroxaban | 250.851 | 377.946 | -6058.51 | -182.751 | 0.473 | 7.579 | 0.35 | 0.527 | -8.451 | -0.255 | 242.979 | 3894.976 |
| Spironolactone | 332.337 | 231.53 | -6609.05 | -244.029 | 0.648 | 18.487 | 0.506 | 0.352 | -10.059 | -0.371 | 279.592 | 7980.986 |
| Trimethoprim | 267.363 | 199.466 | -3237.76 | -248.703 | 0.635 | 10.307 | 0.489 | 0.365 | -5.926 | -0.455 | 189.574 | 3077.209 |
| Valsartan | 351.413 | 289.538 | -7090.68 | -248.743 | 0.606 | 14.85 | 0.478 | 0.394 | -9.64 | -0.338 | 328.067 | 8034.203 |
| Verapamil | 441.347 | 281.926 | -8870.85 | -387.818 | 0.672 | 21.16 | 0.513 | 0.328 | -10.306 | -0.451 | 498.258 | 15677.78 |
| Warfarin | 227.911 | 266.251 | -3895.21 | -204.983 | 0.529 | 7.74 | 0.403 | 0.471 | -6.889 | -0.363 | 169.134 | 2474.394 |

| Drug | WPSA3 | WNSA1 | WNSA2 | WNSA3 | DPSA1 | DPSA2 | DPSA3 | RPCG | WPCS | RNCG | WNCS | SCAA1 |
| --- | --- | --- | --- | --- | --- | --- | --- | --- | --- | --- | --- | --- |
| Allopurinol | 12.863 | 40.054 | -76.008 | -14.73 | 42.985 | 579.42 | 90.371 | 0.254 | 3.495 | 0.241 | 10.611 | -43.296 |
| Amlodipine | 256.603 | 158.636 | -3456.19 | -183.009 | 238.76 | 15145.4 | 632.389 | 0.038 | 1.078 | 0.111 | 1.809 | -70.545 |
| Amoxicillin | 106.141 | 130.374 | -2286.74 | -121.922 | 14.922 | 9088.291 | 440.148 | 0.047 | 0.651 | 0.142 | 0.733 | -162.784 |
| Apixaban | 294.293 | 244.412 | -5401.18 | -204.312 | 173.049 | 17480.44 | 630.334 | 0.038 | 1.08 | 0.104 | 2.184 | -61.302 |
| Aspirin | 48.066 | 70.519 | -554.057 | -58.364 | -21.35 | 2867.966 | 291.568 | 0.102 | 2.44 | 0.307 | 6.295 | -59.113 |
| Atorvastatin | 343.305 | 341.313 | -10134.1 | -290.284 | 120.466 | 26384.93 | 712.989 | 0.028 | 0.115 | 0.084 | 1.077 | -106.698 |
| Cephalexin | 126.59 | 134.632 | -2095.43 | -118.779 | 54.6 | 8512.396 | 448.636 | 0.053 | 0.744 | 0.155 | 2.287 | -126.304 |
| Cinacalcet | 183.376 | 214.132 | -3898.09 | -140.197 | 36.981 | 12254.48 | 480.671 | 0.045 | 0.317 | 0.137 | 1.917 | -4.703 |
| Ciprofloxacin | 131.743 | 125.436 | -1971.26 | -96.304 | 96.5 | 8666.006 | 413.547 | 0.052 | 1.224 | 0.101 | 1.171 | -74.883 |
| Clopidogrel | 123.826 | 140.801 | -1871.79 | -90.974 | 37.894 | 7310.913 | 390.583 | 0.06 | 1.053 | 0.17 | 3.547 | -14.862 |
| Digoxin | 712.617 | 388.545 | -20334 | -466.032 | 457.957 | 59647.63 | 1034.12 | 0.015 | 0.296 | 0.05 | 0.527 | -167.753 |
| Furosemide | 79.542 | 168.001 | -1950.08 | -108.376 | -118.018 | 6078.211 | 358.868 | 0.116 | 0.08 | 0.151 | 1.949 | -92.813 |
| Glipizide | 303.582 | 242.526 | -5945.64 | -197.148 | 185.684 | 19501.08 | 629.485 | 0.055 | 0.091 | 0.097 | 1.871 | -82.35 |
| Lisinopril | 226.25 | 195.936 | -5153.05 | -176.696 | 133.794 | 18316.66 | 578.565 | 0.031 | 0.734 | 0.067 | 1.629 | -145.09 |
| Metformin | 58.475 | 44.422 | -451.826 | -72.374 | 86.977 | 3506.137 | 379.588 | 0.083 | 2.321 | 0.236 | 3.696 | -93.971 |
| Metoprolol | 210.106 | 108.226 | -2172.6 | -143.282 | 264.225 | 12361.01 | 573.915 | 0.04 | 0.476 | 0.127 | 2.02 | -35.242 |
| Phenytoin | 89.261 | 113.705 | -1246.04 | -80.11 | 2.98 | 5242.228 | 354.059 | 0.076 | 2.009 | 0.096 | 0.01 | -44.323 |
| Quinidine | 154.17 | 118.394 | -2288.96 | -128.248 | 150.376 | 10973.09 | 497.59 | 0.041 | 1.122 | 0.119 | 2.496 | -36.975 |
| Riboflavin | 165.604 | 181.043 | -3256.13 | -163.632 | 65.634 | 11428.76 | 518.116 | 0.046 | 1.015 | 0.136 | 2.329 | -136.057 |
| Rivaroxaban | 179.832 | 270.944 | -4343.25 | -131.011 | -39.008 | 11491.71 | 433.602 | 0.052 | 0.426 | 0.1 | 0.048 | -66.997 |
| Spironolactone | 218.363 | 152.128 | -4342.5 | -160.34 | 193.994 | 18755.68 | 576.367 | 0.028 | 0.646 | 0.09 | 0.866 | -73.537 |
| Trimethoprim | 146.09 | 108.99 | -1769.15 | -135.894 | 147.479 | 8869.444 | 516.066 | 0.052 | 1.366 | 0.142 | 2.808 | -23.748 |
| Valsartan | 258.483 | 212.971 | -5215.56 | -182.963 | 156.476 | 18013.36 | 600.156 | 0.034 | 1.064 | 0.103 | 1.286 | -45.309 |
| Verapamil | 379.9 | 242.674 | -7635.8 | -333.824 | 296.923 | 27084.43 | 829.165 | 0.025 | 0.439 | 0.08 | 0.988 | -33.25 |
| Warfarin | 128.86 | 150.537 | -2202.33 | -115.896 | 32.892 | 8271.611 | 432.894 | 0.055 | 1.31 | 0.17 | 3.28 | -48.473 |

| Drug | SCAA2 | SCAA3 | SCDH1 | SCDH2 | SCDH3 | CHDH1 | CHDH2 | CHDH3 | CHAA1 | CHAA2 | CHAA3 | DIPOLE |
| --- | --- | --- | --- | --- | --- | --- | --- | --- | --- | --- | --- | --- |
| Allopurinol | -14.432 | -0.142 | 19.646 | 9.823 | 0.064 | 0.727 | 0.363 | 0.002 | -1.182 | -0.394 | -0.004 | 4.637 |
| Amlodipine | -11.757 | -0.101 | 51.684 | 17.228 | 0.074 | 2.473 | 0.824 | 0.004 | -3.472 | -0.579 | -0.005 | 3.548 |
| Amoxicillin | -27.131 | -0.314 | 41.518 | 8.304 | 0.08 | 4.07 | 0.814 | 0.008 | -5.215 | -0.869 | -0.01 | 6.322 |
| Apixaban | -12.26 | -0.077 | 32.903 | 16.451 | 0.042 | 1.675 | 0.838 | 0.002 | -2.122 | -0.424 | -0.003 | 13.36 |
| Aspirin | -14.778 | -0.162 | 0 | 0 | 0 | 0.792 | 0.792 | 0.002 | -2.286 | -0.572 | -0.006 | 3.321 |
| Atorvastatin | -21.34 | -0.12 | 24.014 | 6.003 | 0.027 | 3.205 | 0.801 | 0.004 | -4.059 | -0.812 | -0.005 | 11.502 |
| Cephalexin | -25.261 | -0.231 | 25.115 | 6.279 | 0.046 | 3.281 | 0.82 | 0.006 | -4.279 | -0.856 | -0.008 | 14.563 |
| Cinacalcet | -4.703 | -0.007 | 5.772 | 5.772 | 0.009 | 0.821 | 0.821 | 0.001 | -0.965 | -0.965 | -0.001 | 7.228 |
| Ciprofloxacin | -18.721 | -0.136 | 19.242 | 9.621 | 0.035 | 1.604 | 0.802 | 0.003 | -2.929 | -0.732 | -0.005 | 14.95 |
| Clopidogrel | -4.954 | -0.027 | 0 | 0 | 0 | 0 | 0 | 0 | -0.683 | -0.228 | -0.001 | 13.955 |
| Digoxin | -11.982 | -0.147 | 65.658 | 10.943 | 0.058 | 4.7 | 0.783 | 0.004 | -8.775 | -0.627 | -0.008 | 30.463 |
| Furosemide | -18.563 | -0.177 | 43.703 | 10.926 | 0.083 | 3.313 | 0.828 | 0.006 | -2.906 | -0.581 | -0.006 | 9.303 |
| Glipizide | -13.725 | -0.104 | 29.057 | 9.686 | 0.037 | 2.478 | 0.826 | 0.003 | -2.994 | -0.499 | -0.004 | 19.812 |
| Lisinopril | -20.727 | -0.208 | 48.804 | 9.761 | 0.07 | 4.057 | 0.811 | 0.006 | -6.331 | -0.904 | -0.009 | 17.264 |
| Metformin | -46.986 | -0.273 | 50.23 | 12.558 | 0.146 | 3.329 | 0.832 | 0.01 | -2.598 | -1.299 | -0.008 | 14.999 |
| Metoprolol | -8.81 | -0.057 | 23.252 | 11.626 | 0.038 | 1.597 | 0.799 | 0.003 | -2.328 | -0.582 | -0.004 | 7.413 |
| Phenytoin | -22.162 | -0.093 | 30.115 | 15.057 | 0.063 | 1.662 | 0.831 | 0.003 | -1.088 | -0.544 | -0.002 | 7.161 |
| Quinidine | -9.244 | -0.065 | 0 | 0 | 0 | 0.779 | 0.779 | 0.001 | -1.501 | -0.375 | -0.003 | 10.729 |
| Riboflavin | -17.007 | -0.214 | 68.96 | 13.792 | 0.109 | 3.992 | 0.798 | 0.006 | -5.582 | -0.698 | -0.009 | 26.647 |
| Rivaroxaban | -13.399 | -0.093 | 6.831 | 6.831 | 0.01 | 0.827 | 0.827 | 0.001 | -2.151 | -0.43 | -0.003 | 2.886 |
| Spironolactone | -18.384 | -0.112 | 0 | 0 | 0 | 0 | 0 | 0 | -1.995 | -0.499 | -0.003 | 3.375 |
| Trimethoprim | -4.75 | -0.043 | 74.5 | 18.625 | 0.136 | 3.346 | 0.837 | 0.006 | -1.41 | -0.282 | -0.003 | 5.687 |
| Valsartan | -7.552 | -0.062 | 26.06 | 13.03 | 0.035 | 1.627 | 0.814 | 0.002 | -2.656 | -0.443 | -0.004 | 11.496 |
| Verapamil | -5.542 | -0.039 | 0 | 0 | 0 | 0 | 0 | 0 | -0.966 | -0.161 | -0.001 | 10.876 |
| Warfarin | -12.118 | -0.086 | 0 | 0 | 0 | 0 | 0 | 0 | -1.797 | -0.449 | -0.003 | 9.193 |

| Drug | DIP2V | HOMO | LUMO | HARDNESS | SC2A | CAA2 | CDA2 | ClogP | tPSA | ACDlogP | ACDlogD1.2 |
| --- | --- | --- | --- | --- | --- | --- | --- | --- | --- | --- | --- |
| Allopurinol | 0.047 | -9.547 | -0.775 | -4.386 | 0.047 | 0.473 | 0.264 | -0.83 | 65.9 | 0.42 | -0.99 |
| Amlodipine | 0.01 | -5.524 | 2.342 | -3.933 | 2.731 | 3.916 | 2.039 | 3.43 | 99.9 | 3.01 | -0.66 |
| Amoxicillin | 0.04 | -7.02 | 1.596 | -4.308 | 2.375 | 5.558 | 3.315 | -1.87 | 133 | 0.88 | -2.15 |
| Apixaban | 0.127 | -6.003 | 1.493 | -3.748 | 2.469 | 1.048 | 1.403 | 1.89 | 108.5 | 4.66 | 4.43 |
| Aspirin | 0.018 | -7.937 | 1.151 | -4.544 | 0.676 | 1.486 | 0.628 | 1.02 | 63.6 | 0.23 | 1.4 |
| Atorvastatin | 0.077 | -6.318 | 1.214 | -3.766 | 3.968 | 3.49 | 2.57 | 4.46 | 110.1 | 3.85 | 3.78 |
| Cephalexin | 0.212 | -6.91 | 1.515 | -4.212 | 1.772 | 4.714 | 2.692 | -1.84 | 112.7 | 0.35 | -2.73 |
| Cinacalcet | 0.045 | -5.315 | 1.298 | -3.307 | 1.969 | 0.931 | 0.674 | 6.35 | 12 | 6.19 | 3.09 |
| Ciprofloxacin | 0.227 | -5.096 | 0.916 | -3.006 | 1.791 | 2.273 | 1.287 | -0.73 | 72.9 | 1.63 | -1.48 |
| Clopidogrel | 0.203 | -5.396 | 1.137 | -3.267 | 1.285 | 0.305 | 0 | 4.21 | 29.5 | 2.58 | -0.33 |
| Digoxin | 0.416 | -4.293 | 2.423 | -3.358 | 9.612 | 7.034 | 3.682 | 1.42 | 203.1 | 1.29 | 1.29 |
| Furosemide | 0.096 | -7.041 | 0.513 | -3.777 | 1.029 | 1.973 | 2.745 | 1.9 | 118.7 | 2.3 | 2.3 |
| Glipizide | 0.279 | -5.05 | 0.572 | -2.811 | 3.022 | 1.585 | 2.047 | 2.57 | 129.1 | 1.88 | 1.51 |
| Lisinopril | 0.229 | -5.455 | 2.083 | -3.769 | 3.973 | 6.797 | 3.292 | -1.82 | 133 | 3.47 | -0.42 |
| Metformin | 0.428 | -6.129 | 3.239 | -4.684 | 1.2 | 3.382 | 2.771 | -1.63 | 89 | -1.25 | -4.17 |
| Metoprolol | 0.053 | -4.994 | 2.423 | -3.708 | 2.618 | 2.107 | 1.276 | 1.49 | 50.7 | 1.63 | -1.47 |
| Phenytoin | 0.063 | -7.559 | 1.58 | -4.569 | 1.004 | 0.592 | 1.38 | 2.09 | 58.2 | 1.42 | 1.42 |
| Quinidine | 0.111 | -4.938 | 1.613 | -3.275 | 2.634 | 1.113 | 0.606 | 2.79 | 45.1 | 2.82 | -1.28 |
| Riboflavin | 0.635 | -6.13 | 0.723 | -3.426 | 2.036 | 4.519 | 3.19 | -0.73 | 155.1 | 0 | 0 |
| Rivaroxaban | 0.007 | -6.315 | -0.465 | -2.925 | 1.434 | 1.065 | 0.683 | 2.39 | 88.2 | 1.71 | 1.47 |
| Spironolactone | 0.009 | -5.46 | 1.528 | -3.494 | 4.96 | 1.034 | 0 | 2.65 | 60.4 | 3.15 | 3.15 |
| Trimethoprim | 0.034 | -6.194 | 2.039 | -4.117 | 1.929 | 0.574 | 2.799 | 0.98 | 104.5 | 0.59 | -1.91 |
| Valsartan | 0.096 | -5.067 | 0.653 | -2.86 | 3.261 | 1.652 | 1.325 | 4.87 | 106.7 | 4.93 | 4.74 |
| Verapamil | 0.076 | -4.459 | 2.372 | -3.416 | 4.601 | 0.359 | 0 | 4.47 | 64 | 4.02 | 0.92 |
| Warfarin | 0.085 | -6.93 | 0.373 | -3.651 | 1.514 | 0.84 | 0 | 1.97 | 60.4 | 1.99 | 1.99 |

| Drug | ACDlogD3 | ACDlogD4.5 | ACDlogD5.9 | ACDlogD6.8 | #amine | #amidine | #acid | #amide | #rotor | #in34 |
| --- | --- | --- | --- | --- | --- | --- | --- | --- | --- | --- |
| Allopurinol | -0.56 | -0.55 | -0.55 | -0.55 | 0 | 0 | 0 | 0 | 0 | 0 |
| Amlodipine | -0.12 | 0 | 0.26 | 0.94 | 1 | 0 | 0 | 0 | 8 | 0 |
| Amoxicillin | -1.69 | -1.61 | -1.68 | -1.96 | 1 | 0 | 1 | 2 | 6 | 4 |
| Apixaban | 4.65 | 4.66 | 4.66 | 4.66 | 0 | 0 | 0 | 0 | 2 | 0 |
| Aspirin | 1.28 | 0.34 | -0.95 | -1.53 | 0 | 0 | 1 | 0 | 2 | 0 |
| Atorvastatin | 3.82 | 3.36 | 2.13 | 1.26 | 0 | 0 | 1 | 0 | 12 | 0 |
| Cephalexin | -2.34 | -2.11 | -2.19 | -2.56 | 1 | 0 | 1 | 1 | 5 | 4 |
| Cinacalcet | 3.09 | 3.1 | 3.3 | 3.87 | 1 | 0 | 0 | 0 | 6 | 0 |
| Ciprofloxacin | -1.47 | -1.4 | -0.81 | -0.38 | 1 | 0 | 1 | 0 | 1 | 3 |
| Clopidogrel | 1.02 | 2.25 | 2.56 | 2.58 | 1 | 0 | 0 | 0 | 3 | 0 |
| Digoxin | 1.29 | 1.29 | 1.29 | 1.29 | 0 | 0 | 0 | 0 | 12 | 0 |
| Furosemide | 2.01 | 0.81 | -0.4 | -0.76 | 0 | 0 | 1 | 0 | 6 | 0 |
| Glipizide | 1.87 | 1.78 | 1.02 | 0.33 | 0 | 0 | 0 | 1 | 7 | 0 |
| Lisinopril | 0.5 | 0.94 | 0.94 | 0.75 | 2 | 0 | 2 | 1 | 13 | 0 |
| Metformin | -3.45 | -3.26 | -3.25 | -3.25 | 0 | 2 | 0 | 0 | 2 | 0 |
| Metoprolol | -1.47 | -1.46 | -1.36 | -0.95 | 1 | 0 | 0 | 0 | 10 | 0 |
| Phenytoin | 1.42 | 1.42 | 1.42 | 1.42 | 0 | 0 | 0 | 0 | 0 | 0 |
| Quinidine | -1.22 | -0.67 | -0.12 | 0.45 | 1 | 0 | 0 | 0 | 5 | 0 |
| Riboflavin | 0 | 0 | 0 | 0 | 0 | 0 | 0 | 0 | 9 | 0 |
| Rivaroxaban | 1.71 | 1.71 | 1.71 | 1.71 | 0 | 0 | 0 | 0 | 3 | 0 |
| Spironolactone | 3.15 | 3.15 | 3.15 | 3.15 | 0 | 0 | 0 | 0 | 2 | 0 |
| Trimethoprim | -1.89 | -1.63 | -0.57 | 0.15 | 0 | 0 | 0 | 0 | 7 | 0 |
| Valsartan | 4.7 | 3.15 | 0.84 | 0.26 | 0 | 0 | 1 | 1 | 10 | 0 |
| Verapamil | 0.92 | 0.94 | 1.24 | 1.9 | 1 | 0 | 0 | 0 | 14 | 0 |
| Warfarin | 1.99 | 1.99 | 1.99 | 1.99 | 0 | 0 | 0 | 0 | 4 | 0 |

# Supplementary Table SII. QSPR input data and variables

| **Drug** | **SGF** | **SGF**  **Pre-**  **dicted** | **AB** | **AB**  **Pre-**  **dicted** | **SIF** | **SIF Predicted** | **QPlogPw** | **IP.eV** | **EA.eV** | **SAAA1** | **X.**  **non-con** | **X.**  **amide** |
| --- | --- | --- | --- | --- | --- | --- | --- | --- | --- | --- | --- | --- |
| Allopurinol | 85.7 | 86.8 | 90.1 | 91.7 | 94.2 | 93.9 | 9.814 | 9.547 | 0.775 | 107.292 | 0 | 0 |
| Amlodipine | 10.8 | 35.7 | 36.6 | 55 | 13.1 | 42.2 | 10.601 | 8.425 | 0.423 | 77.982 | 1 | 0 |
| Amoxicillin | ND | ND | 99.2 | 111.7 | 99.4 | 112.1 | 21.458 | 9.232 | 0.587 | 190.044 | 4 | 2 |
| Apixaban | 75.6 | 70.9 | 97.3 | 76.5 | 97.8 | 68.5 | 17.104 | 8.856 | 0.552 | 131.016 | 6 | 0 |
| Aspirin | 100.7 | 94.7 | 99.8 | 100.6 | 99.9 | 105.2 | 8.067 | 9.868 | 0.621 | 101.381 | 0 | 0 |
| Atorvastatin | 91.2 | 62.4 | 93.3 | 83.5 | 101.1 | 76 | 14.804 | 8.591 | 0.377 | 134.833 | 0 | 0 |
| Cephalexin | 88.7 | 79.5 | 95 | 99.6 | 104.4 | 101.6 | 16.599 | 9.421 | 0.875 | 153.7 | 3 | 1 |
| Cinacalcet | 13.4 | 26.5 | 19.3 | 27.4 | 18.3 | 14.3 | 5.694 | 8.605 | 0.787 | 4.875 | 0 | 0 |
| Ciprofloxacin | 18 | 34.7 | 24.8 | 58.7 | 6.9 | 48.6 | 9.952 | 8.742 | 0.821 | 108.283 | 7 | 0 |
| Clopidogrel | 66 | 43.3 | ND | ND | ND | ND | 6.269 | 9.058 | 0.389 | 36.133 | 3 | 0 |
| Digoxin | ND | ND | 109.3 | 108.6 | 103.4 | 103.1 | 32.638 | 10.258 | 0.428 | 225.097 | 33 | 0 |
| Furosemide | 67.3 | 75.7 | 94.5 | 88.2 | 79.1 | 86.6 | 14.01 | 8.944 | 0.871 | 144.229 | 0 | 0 |
| Glipizide | 72.9 | 84.4 | 96.5 | 102 | 98.5 | 106.8 | 15.084 | 9.798 | 1.073 | 155.062 | 6 | 1 |
| Lisinopril | 77.9 | 90.5 | 100.7 | 119.4 | 98.5 | 118.3 | 20.147 | 9.398 | -0.046 | 170.919 | 4 | 1 |
| Metformin | 48.9 | 50.8 | 81.8 | 75.7 | 80.2 | 61.2 | 12.109 | 8.573 | -0.589 | 73.008 | 0 | 0 |
| Metoprolol | 71.7 | 54.9 | 85.9 | 73 | 68.6 | 62.6 | 8.773 | 8.951 | -0.256 | 54.311 | 0 | 0 |
| Phenytoin | 83.4 | 99.8 | 90.2 | 95.9 | 92.8 | 97.4 | 10.24 | 9.836 | 0.289 | 81.047 | 1 | 0 |
| Quinidine | 12.6 | 21.5 | 43.1 | 38.2 | 24.3 | 23.6 | 8.535 | 8.58 | 0.756 | 62.976 | 7 | 0 |
| Riboflavin | 95.6 | 94.6 | ND | ND | 96.5 | 96.7 | 21.03 | 8.745 | 1.39 | 199.937 | 0 | 0 |
| Rivaroxaban | 71.9 | 64.9 | 92.9 | 72 | 95.1 | 67.6 | 15.164 | 8.847 | 1.224 | 145.245 | 5 | 0 |
| Spironolactone | 78.7 | 66.2 | 98.6 | 90 | 96.8 | 87 | 9.009 | 9.923 | 0.553 | 134.068 | 16 | 0 |
| Trimethoprim | 36.3 | 40.9 | 55.3 | 60.3 | 28.4 | 45.4 | 12.081 | 8.362 | -0.072 | 70.247 | 0 | 0 |
| Valsartan | 86.4 | 71.5 | 101.4 | 94.7 | 98 | 94.6 | 15.14 | 9.236 | 0.666 | 131.855 | 0 | 1 |
| Verapamil | 51.7 | 36.8 | 88.6 | 80.1 | 77.9 | 70.8 | 6.537 | 8.754 | -0.028 | 96.776 | 0 | 0 |
| Warfarin | 66.3 | 84.6 | 92.5 | 83.9 | 97.3 | 86.4 | 9.617 | 9.564 | 1.065 | 102.664 | 1 | 0 |
